# The Duties of a Dentist to Himself and Others

**Published:** 1886-03

**Authors:** G. L. Curtis

**Affiliations:** Syracuse, N. Y.


### THE DUTIES OF A DENTIST TO HIMSELF AND OTHERS.

BY G. L. CURTIS, D. D., SYRACUSE, N. Y.

ANNUAL ADDRESS BEFORE THE UNION MEETING OF THE FIFTH AND SIXTH DISTRICT DENTAL SOCIETIES OF THE STATE OF NEW YORK AT BINGHAMTON.

It is my purpose briefly to sketch a few things observed in everyday life, and to throw out to the younger dentists a few suggestions which, if observed, cannot but aid him in starting in his preferred calling.

Having selected a school of high grade and taken therefrom a diploma admitting you to practice in any part of America, if already a location has not been selected you turn your efforts to this end. Having made your selection, the question may arise whether you will pull alone in the race or follow after the older practitioner, your neighbor next door, and whether or not you will make his acquaintance. I can only from observation state that it is better under all circumstances to be on good terms with the older practitioner, through whose efforts you have attained your knowledge, and of whose counsel you, as a progressive dentist, cannot afford to deprive yourself. There is but little pleasure or satisfaction in isolation, and it is only selfishness that prompts such action. Therefore, be respectful to your superior and try to be as he, a benefactor and blessing to those who seek your services, at the same time remembering that it is to you a compliment, and not to him, if the friendship is mutual.

We all have some aim in life. Some strive for excellence in their vocation, some for position, and some to obtain wealth. The first resolution is a noble one, and you and I cannot aim too high in compassing it. It is the only way to success. In every way prepare yourself to meet the demands made upon you, and in all your efforts let the last be the best. Take the latest and best literature of the day, and carefully study it. (I fear this suggestion might to advantage be adopted by many of our older practitioners.) Reading alone, however, can accomplish only a part. Sift thoroughly the good from all, and put the best into practice. Be conscientious in all your operations, and never allow a piece of work to go from your office until it is finished to your own satisfaction. Please yourself, and your patient cannot but approve your services. Make good your losses to your patients, and never lose the opportunity to make good a failure without expense to him, by repairing or doing over a piece of work which did not at first meet with success. In giving advice never say a piece of work cannot be done because you are not equal to it, when you know another man can do it. Either learn how to do this yourself, or refer the inquirer where it can be accomplished.

Great caution should be exercised in extracting, and I believe it should be made a criminal offense to remove a tooth when it can and should be saved, even though the patient demand it.

In these days, when everyone is taught and should know the value of teeth, the man who gives way to such requests belittles himself in the mind of his patient, and leaves a stain upon his own conscience for life, for teeth are to man what the mill is to the grain.

Strive to be a credit to the community in which you live, as well as a guide to others. There is no better way to gain position in dentistry or in society than by being a *man*, for manliness, above all things, commands respect. Be true to yourself and all with whom you come in contact. Never shrink from duty. Maintain dignity in your office, and never, above all things, lose your head.

Wealth is the least of all for which we in this life should exclusively labor, for a more selfish aim one cannot possess. Always thinking of the comfort and welfare of others, the dentist, above all men, should be the last unduly to strive for riches, and if you will pause for a moment to consider that never, as yet, has a dentist become rich from his own labors, you will discard this object in life and aim for a nobler one. What is wanted by the American people is an educated, energetic, wide-awake dentist, and to keep abreast with the times of to-day you must, at least, be better advised than those you are called upon to serve. We do not live in those good olden times, ages ago, when haste was rarely considered, and time was not associated with daily life and the duties of existence. When we consider the way in which time was once spent in all and everything that was done, one can readily account for the long and easy life, and the methodical ways in which their work was accomplished. This condition of affairs is even yet prominent in some parts of Europe, and they still hold fast to the opinion that anything done by the active, energetic American is a kind of "Yankee notion," of short duration, and not worthy of note. Hence the conservative way of the English, who are of all nations perhaps the last to accept a new idea or to put into practice a new theory. It is the American, full of life, of energy, always ready to meet all emergencies, who knows not the definition of the word "impossible," and who has stamped in his very face that look so forcibly described by Herbert Spencer as a "do or die" expression, who has brought dentistry up to its present high standard and helped to make America what she is to-day.

In society work it should be our highest purpose to elevate the standard by doing all in our power for the good of the society and one another. Attend regularly the stated meetings, remembering that you are the loser when you remain away, and that it cannot be made up to you by money. We often hear the young man remark: "Oh, I cannot afford to attend these meetings, I would lose so much time." This is a great mistake, which you may not see till it is too late. Bring the best material to the front and welcome the efforts of the younger members, thus encouraging them to do greater things.

Now for a word to the old practitioners. Conscious of the fact that he was once young, he should in every way try to encourage and aid his followers in the right direction, for it is good to work together in unison, and what is more gratifying than to see the old and young in council. We too often, however, find a well established dentist like the cat, ready to pounce upon the harmless young canine the instant it comes into its vicinity, and never to show him any quarter till he proves himself master of the situation, after living down the impositions thrust upon him in every conceivable way. True to the conception of a clerical friend, "sanctification sometimes means scorchification," and in order to be sufficiently tempered to meet cheerfully the taunts and slurs of the jealous competitor, who always has a bland smile of pretended friendship for you, it seems necessary to pass through this furnace of friendly hatred in order to get a peep into the inward workings of those who, by chance, perhaps, have slipped into dentistry, and feel that they alone wish to be recognized as the dentist, and who try to maintain this position by depreciating the works and abilities of their neighbor, who perhaps in more ways than one is his equal or superior. This is not only a wrong course to adopt, but it is unprofessional and unbecoming a gentleman, and it is only a question of time, among the better class of dentists at least, when this will be a thing of the past.

